# Hydrogen bonding in the crystal structure of the molecular salt of pyrazole–pyrazolium picrate

**DOI:** 10.1107/S2056989016008215

**Published:** 2016-05-27

**Authors:** Ping Su, Xue-gang Song, Ren-qiang Sun, Xing-man Xu

**Affiliations:** a College of Chemistry, Central China Normal University, Wuhan 430079, People’s Republic of China

**Keywords:** crystal structure, organic salt, pyrazole, picric acid, hydrogen bonding

## Abstract

The asymmetric unit of the title organic salt [systematic name: 1*H*-pyrazol-2-ium 2,4,6-tri­nitro­phenolate–1*H*-pyrazole (1/1)], H(C_3_H_4_N_2_)_2_^+^·C_6_H_2_N_3_O_7_^−^, consists of one picrate anion and one hydrogen-bonded dimer of a pyrazolium monocation. The H atom involved in the dimer N—H⋯N hydrogen bond is disordered over both symmetry-unique pyrazole mol­ecules with occupancies of 0.52 (5) and 0.48 (5). In the crystal, the component ions are linked into chains along [100] by two different bifurcated N—H⋯(O,O) hydrogen bonds. In addition, weak C—H⋯O hydrogen bonds link inversion-related chains, forming columns along [100].

## Abstract 2

A 2:1 organic salt formed from pyrazole and picric acid was obtained from methanol solution. In the crystal, N—H⋯O hydrogen bonds give rise to a hydrogen-bonded chain along [100]. Adjacent [100] chains are linked by a weak C—H⋯O inter­action.

## Chemical context   

Research inter­est on co-crystals or organic complex salts in recent years has been prompted by their potential utilization in the pharmaceutical industry (Blagden *et al.*, 2014 ▸; Duggirala *et al.*, 2016 ▸). Imidazole and pyrazole derivatives are often used as co-crystallized pharmaceutical ingredients (Shimpi *et al.*, 2014 ▸). Our investigations involve studies of weak inter­molecular inter­actions in co-crystallized compounds. As part of our continuing study on organic salts formed by imidazole derivatives and picric acid (Song *et al.*, 2016 ▸; Su *et al.*, 2008 ▸), we report herein the crystal structure of the title compound (I).
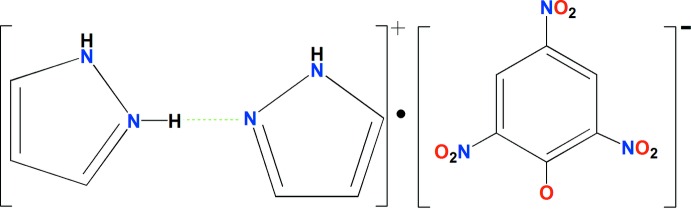


## Structural commentary   

**Figure 1:**
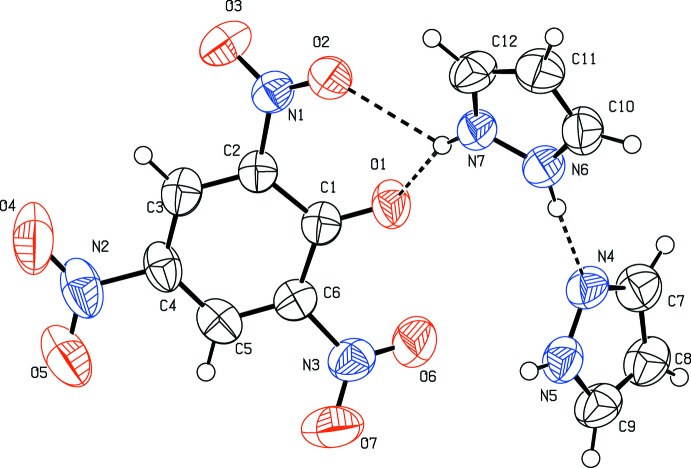
The mol­ecular structure of (I), showing the atom-numbering scheme. Displacement ellipsoids are drawn at the 50% probability level. Hydrogen bonds are shown as dashed lines. Only one orientation of the disordered N—H⋯N hydrogen bond is shown.

The asymmetric unit of the title compound is shown in Fig. 1 ▸. It consists of one picrate anion and two pyrazole mol­ecules, which are connected by an N—H⋯N hydrogen bond (Table 1 ▸), forming a dimeric pyrazolium monocation. The H atom of the hydrogen bond is disordered over both pyrazole mol­ecules. In the dimeric monocation, the two pyrazole rings form a dihedral angle of 74.6 (1)°. In the anion, the C—O_phenol_ bond [1.257 (3)Å] is shorter by *ca* 0.05Å than an average C—O single bond in a neutral picric acid mol­ecule [1.308 (2)Å] calculated statistically by analysis of a CSD search (Groom *et al.*, 2016 ▸; Allen, 2002 ▸). The C1—C2 [1.438 (4)Å] and C1—C6 [1.449 (4)Å] bonds are significantly longer than the other four benzene C-C bonds [1.367 (4)–1.380 (4)Å]. The C2—C1—C6 [111.9 (2)°] angle is smaller than the ideal value of 120° for a regular hexa­gon and the other five benzene inner angles of 119.0 (3)–124.4 (3). All variations of bond lengths and angles demonstrate that the negative charge on the phenol oxygen atom is delocalized over the aromatic ring, giving double-bond character for the C1—O1 bond due to the electron-withdrawing effect of the three nitro groups. This is similar to what is observed in some picrate-containing analogs (Zakharov *et al.*, 2015 ▸; Gomathi & Kalaivani, 2015 ▸). The mean planes of the nitro groups in the anion, are twisted from the benzene ring by dihedral angles of 30.8 (2), 4.8 (3)° and 27.2 (4)° for N1/O2/O3, N2/O4/O5 and N3/O6/O7, respectively. The two *ortho*-nitro groups are twisted out of the benzene ring to a greater extent than the *para*-nitro group. This is most likely due to the steric hindrance between the *ortho*-nitro groups and the phenolic oxygen atom.

## Supra­molecular features   

**Figure 2:**
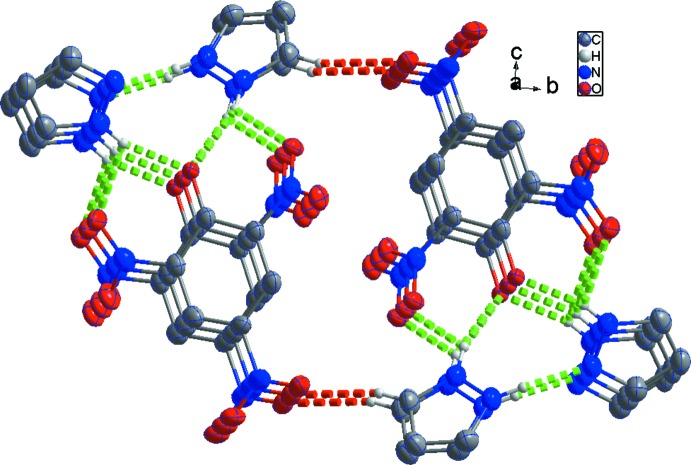
Part of the crystal structure of (I), showing the formation of hydrogen-bonded columns along [100]. For clarity, H atoms not involved in the motif have been omitted. Green and red dashed lines indicate the N—H⋯O hydrogen bonds and weak C—H⋯O hydrogen bonds, respectively.

In the crystal of (I), the component ions are linked into a chain along [100] by N—H⋯O hydrogen bonds (Table 1 ▸, Fig. 2 ▸). In addition, inversion-related chains are connected by a weak C12—H12⋯O4 (−*x*, −*y* + 2, −*z* + 1) hydrogen bond, forming columns along [100]. A short O3_(nitro)_⋯O3_(nitro)_ (−1 − *x*, 2 − *y*, 1 − *z*) distance of 2.913 (2) Å is also observed (Spek, 2009 ▸). Although the benzene and pyrazolium rings are stacked in a parallel fashion, no significant π–π inter­actions exist between them (Janiak, 2000 ▸). This could be attributed to the deficient π-electron nature resulting from the electron-withdrawing effects of the nitro groups.

## Database survey   

A search of the Cambridge Structural Database (CSD Version 5.37 plus one update; Groom *et al.*, 2016 ▸) indicates there are some analogs prepared from picric acid and pyrazole derivatives, *viz*. SASKII, SASLAB, SASKUU, SASLUB (Singh *et al.*, 2012 ▸) and SASKII01 (Dhanabal *et al.*, 2013 ▸). A similar solvated organic adduct, C_5_H_9_N_2_^+^·C_6_H_2_N_3_O_7_^−^ (SASKII; Singh *et al.*, 2012 ▸) indicates that the solvent used for the crystallization process can affect the final product in which the ratio of component ions are different.

## Synthesis and crystallization   

Pyrazole (20.0 mmol, 136.0 mg) and picric acid (10. 0 mmol, 230.0mg) were dissolved in a 2:1 molar ratio in 95% methanol (50.0 ml). The mixture was stirred for an hour at 323 K and then cooled to room temperature and filtered. The resulting yellow solution was kept in air for two weeks. Needle-like yellow crystals of (I) suitable for single-crystal X-ray diffraction analysis were grown by slow evaporation of the solution. The crystals were separated by filtration (yield, 60%, *ca* 0.22 g).

## Refinement   

Crystal data, data collection and structure refinement details are summarized in Table 2 ▸. H atoms bonded to C atoms were positioned geometrically with C—H = 0.93 Å (aromatic) and refined in a riding-model approximation with *U*_iso_(H) = 1.2*U*_eq_(C). H atoms bonded to N atoms were refined with a constraint of *d*_N—H_ = 0.86 (1) Å and *U*_iso_(H) = 1.2*U*_eq_(N). Atoms H4*A* and H6*A* were found in difference Fourier maps and refined as disordered using the PART command (Sheldrick, 2015 ▸). The final site occupancies of the two hydrogen-atom components were 0.52 (1):0.48 (1) for H6*A* and H4*A*, respectively.

## Supplementary Material

Crystal structure: contains datablock(s) global, I. DOI: 10.1107/S2056989016008215/lh5810sup1.cif

Structure factors: contains datablock(s) I. DOI: 10.1107/S2056989016008215/lh5810Isup2.hkl

Supporting information file. DOI: 10.1107/S2056989016008215/lh5810Isup3.cml

CCDC reference: 1480969

Additional supporting information:  crystallographic information; 3D view; checkCIF report

## Figures and Tables

**Table 1:** Hydrogen-bond geometry (Å, °)

*D*—H⋯*A*	*D*—H	H⋯*A*	*D*⋯*A*	*D*—H⋯*A*
N4—H4*A*⋯N6	0.86 (1)	1.81 (1)	2.663 (3)	173 (7)
N5—H5*A*⋯O1^i^	0.87 (1)	1.95 (1)	2.789 (3)	163 (3)
N5—H5*A*⋯O6^i^	0.87 (1)	2.42 (3)	2.961 (4)	121 (3)
N6—H6*A*⋯N4	0.86 (1)	1.81 (1)	2.663 (3)	174 (7)
N7—H7*A*⋯O1	0.86 (1)	2.04 (2)	2.864 (3)	160 (3)
N7—H7*A*⋯O2	0.86 (1)	2.29 (3)	2.841 (3)	122 (3)
C12—H12⋯O4^ii^	0.93	2.61	3.512 (5)	165

Symmetry codes: (i) 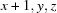; (ii) 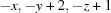.

**Table 2:** Experimental details

Crystal data	
Chemical formula	C_3_H_5_N_2_^+^·C_6_H_2_N_3_O_7_^−^·C_3_H_4_N_2_
*M*_r_	365.28
Crystal system, space group	Monoclinic, *P*2_1_/*c*
Temperature (K)	298
*a*, *b*, *c* (Å)	4.2447 (14), 16.950 (5), 21.839 (7)
β (°)	92.029 (6)
*V* (Å^3^)	1570.3 (9)
*Z*	4
Radiation type	Mo *K*α
μ (mm^−1^)	0.13
Crystal size (mm)	0.45 × 0.06 × 0.04

Data collection	
Diffractometer	Bruker SMART CCD
Absorption correction	Multi-scan (*SADABS*; Sheldrick, 1996 ▸)
*T*_min_, *T*_max_	0.736, 0.875
No. of measured, independent and observed [*I* > 2σ(*I*)] reflections	12038, 3086, 1787
*R*_int_	0.050
(sin θ/λ)_max_ (Å^−1^)	0.617

Refinement	
*R*[*F* ^2^ > 2σ(*F* ^2^)], *wR*(*F* ^2^), *S*	0.057, 0.157, 0.98
No. of reflections	3086
No. of parameters	248
No. of restraints	4
H-atom treatment	H atoms treated by a mixture of independent and constrained refinement
Δρ_max_, Δρ_min_ (e Å^−3^)	0.18, −0.16

Computer programs: *SMART* and *SAINT* (Bruker, 2001 ▸), *SHELXS* and *SHELXTL* (Sheldrick, 2008 ▸), *SHELXL2014* (Sheldrick, 2015 ▸) and *DIAMOND* (Brandenburg, 2006 ▸).

## supplementary crystallographic information

### Crystal data

**Table:**

C_3_H_5_N_2_^+^·C_6_H_2_N_3_O_7_^−^·C_3_H_4_N_2_	*F*(000) = 752
*M**_r_* = 365.28	*D*_x_ = 1.545 Mg m^−^^3^
Monoclinic, *P*2_1_/*c*	Mo *K*α radiation, λ = 0.71073 Å
*a* = 4.2447 (14) Å	Cell parameters from 1735 reflections
*b* = 16.950 (5) Å	θ = 2.4–20.5°
*c* = 21.839 (7) Å	µ = 0.13 mm^−^^1^
β = 92.029 (6)°	*T* = 298 K
*V* = 1570.3 (9) Å^3^	Needle, yellow
*Z* = 4	0.45 × 0.06 × 0.04 mm

### Data collection

**Table:**

Bruker SMART CCD diffractometer	1787 reflections with *I* > 2σ(*I*)
φ and ω scans	*R*_int_ = 0.050
Absorption correction: multi-scan (*SADABS*; Sheldrick, 1996)	θ_max_ = 26.0°, θ_min_ = 1.5°
*T*_min_ = 0.736, *T*_max_ = 0.875	*h* = −5→5
12038 measured reflections	*k* = −20→20
3086 independent reflections	*l* = −26→24

### Refinement

**Table:**

Refinement on *F*^2^	4 restraints
Least-squares matrix: full	H atoms treated by a mixture of independent and constrained refinement
*R*[*F*^2^ > 2σ(*F*^2^)] = 0.057	*w* = 1/[σ^2^(*F*_o_^2^) + (0.0698*P*)^2^ + 0.3803*P*] where *P* = (*F*_o_^2^ + 2*F*_c_^2^)/3
*wR*(*F*^2^) = 0.157	(Δ/σ)_max_ < 0.001
*S* = 0.98	Δρ_max_ = 0.18 e Å^−^^3^
3086 reflections	Δρ_min_ = −0.16 e Å^−^^3^
248 parameters	

### Special details

**Table:**

Geometry. All esds (except the esd in the dihedral angle between two l.s. planes) are estimated using the full covariance matrix. The cell esds are taken into account individually in the estimation of esds in distances, angles and torsion angles; correlations between esds in cell parameters are only used when they are defined by crystal symmetry. An approximate (isotropic) treatment of cell esds is used for estimating esds involving l.s. planes.

### Fractional atomic coordinates and isotropic or equivalent isotropic displacement parameters (Å^2^)

**Table:**

	*x*	*y*	*z*	*U*_iso_*/*U*_eq_	Occ. (<1)
C1	0.2577 (7)	0.78567 (15)	0.50270 (13)	0.0502 (7)	
C2	0.0904 (7)	0.85715 (15)	0.51570 (12)	0.0478 (7)	
C3	0.0496 (7)	0.88621 (16)	0.57336 (13)	0.0565 (8)	
H3	−0.0667	0.9320	0.5789	0.068*	
C4	0.1825 (8)	0.84691 (17)	0.62309 (13)	0.0577 (8)	
C5	0.3503 (7)	0.77808 (17)	0.61557 (13)	0.0573 (8)	
H5	0.4423	0.7525	0.6494	0.069*	
C6	0.3802 (7)	0.74780 (15)	0.55807 (13)	0.0506 (7)	
C7	0.7321 (8)	0.56590 (19)	0.33757 (15)	0.0683 (9)	
H7	0.5902	0.5601	0.3045	0.082*	
C8	0.8600 (8)	0.50504 (18)	0.37140 (16)	0.0689 (9)	
H8	0.8229	0.4514	0.3660	0.083*	
C9	1.0517 (8)	0.53966 (19)	0.41426 (15)	0.0679 (9)	
H9	1.1731	0.5137	0.4443	0.082*	
C10	0.8031 (8)	0.80992 (19)	0.26399 (14)	0.0675 (9)	
H10	0.9374	0.7862	0.2367	0.081*	
C11	0.6789 (9)	0.88402 (19)	0.25746 (15)	0.0698 (9)	
H11	0.7114	0.9197	0.2260	0.084*	
C12	0.4981 (8)	0.89415 (18)	0.30687 (15)	0.0648 (9)	
H12	0.3809	0.9388	0.3157	0.078*	
N1	−0.0457 (6)	0.90430 (13)	0.46540 (12)	0.0532 (6)	
N2	0.1422 (9)	0.87879 (19)	0.68408 (13)	0.0815 (9)	
N3	0.5497 (7)	0.67352 (16)	0.55396 (14)	0.0645 (7)	
N4	0.8395 (7)	0.63381 (15)	0.35881 (12)	0.0613 (7)	
H4A	0.808 (16)	0.6815 (15)	0.346 (3)	0.074*	0.48 (5)
N5	1.0360 (7)	0.61758 (14)	0.40591 (12)	0.0617 (7)	
H5A	1.133 (7)	0.6554 (14)	0.4254 (13)	0.074*	
N6	0.7043 (7)	0.77681 (14)	0.31469 (12)	0.0600 (7)	
H6A	0.761 (15)	0.7321 (19)	0.330 (3)	0.072*	0.52 (5)
N7	0.5183 (6)	0.82915 (14)	0.34019 (11)	0.0553 (6)	
H7A	0.434 (7)	0.8196 (18)	0.3747 (8)	0.066*	
O1	0.2919 (6)	0.75921 (11)	0.44959 (9)	0.0668 (6)	
O2	0.0820 (5)	0.90477 (13)	0.41655 (9)	0.0709 (7)	
O3	−0.2780 (5)	0.94352 (13)	0.47569 (11)	0.0756 (7)	
O4	−0.0220 (9)	0.93651 (17)	0.68985 (11)	0.1281 (13)	
O5	0.2766 (8)	0.84518 (17)	0.72727 (12)	0.1047 (10)	
O6	0.4897 (7)	0.62868 (15)	0.51168 (12)	0.0976 (9)	
O7	0.7405 (7)	0.65737 (15)	0.59511 (13)	0.0968 (9)	

### Atomic displacement parameters (Å^2^)

**Table:**

	*U*^11^	*U*^22^	*U*^33^	*U*^12^	*U*^13^	*U*^23^
C1	0.0571 (19)	0.0411 (15)	0.0529 (18)	−0.0044 (13)	0.0093 (14)	0.0021 (13)
C2	0.0536 (18)	0.0405 (14)	0.0496 (17)	−0.0054 (13)	0.0070 (14)	0.0018 (12)
C3	0.069 (2)	0.0407 (15)	0.0609 (19)	−0.0060 (14)	0.0160 (16)	0.0012 (14)
C4	0.083 (2)	0.0481 (17)	0.0431 (17)	−0.0152 (16)	0.0113 (16)	−0.0028 (13)
C5	0.067 (2)	0.0531 (17)	0.0517 (18)	−0.0163 (15)	0.0001 (15)	0.0063 (14)
C6	0.0516 (18)	0.0417 (15)	0.0586 (19)	−0.0056 (13)	0.0047 (14)	0.0059 (13)
C7	0.081 (2)	0.0542 (19)	0.070 (2)	0.0002 (17)	0.0032 (18)	0.0008 (16)
C8	0.082 (2)	0.0443 (17)	0.081 (2)	0.0001 (17)	0.011 (2)	0.0028 (17)
C9	0.079 (2)	0.0543 (19)	0.072 (2)	0.0136 (17)	0.0130 (19)	0.0148 (16)
C10	0.082 (2)	0.064 (2)	0.057 (2)	0.0059 (18)	0.0108 (17)	−0.0026 (16)
C11	0.084 (2)	0.062 (2)	0.064 (2)	0.0043 (18)	0.0063 (18)	0.0165 (16)
C12	0.071 (2)	0.0494 (17)	0.074 (2)	0.0102 (15)	0.0029 (18)	0.0093 (16)
N1	0.0543 (16)	0.0428 (13)	0.0625 (17)	−0.0019 (12)	0.0015 (13)	−0.0003 (12)
N2	0.136 (3)	0.0584 (18)	0.0513 (18)	−0.0198 (18)	0.0169 (18)	−0.0022 (15)
N3	0.0695 (19)	0.0586 (16)	0.0659 (18)	0.0079 (14)	0.0087 (15)	0.0137 (15)
N4	0.080 (2)	0.0445 (15)	0.0593 (17)	0.0116 (14)	0.0079 (15)	0.0077 (13)
N5	0.075 (2)	0.0482 (16)	0.0624 (18)	0.0046 (13)	0.0103 (15)	−0.0023 (13)
N6	0.0765 (19)	0.0439 (14)	0.0593 (17)	0.0071 (13)	−0.0008 (14)	0.0020 (13)
N7	0.0655 (17)	0.0490 (14)	0.0513 (15)	0.0082 (12)	0.0033 (12)	0.0009 (12)
O1	0.1065 (18)	0.0453 (11)	0.0493 (13)	0.0069 (11)	0.0120 (12)	0.0007 (9)
O2	0.0857 (17)	0.0755 (15)	0.0519 (14)	0.0201 (12)	0.0063 (12)	0.0082 (11)
O3	0.0627 (15)	0.0675 (14)	0.0969 (18)	0.0187 (12)	0.0090 (13)	0.0079 (12)
O4	0.244 (4)	0.0713 (18)	0.0722 (18)	0.027 (2)	0.046 (2)	−0.0084 (14)
O5	0.153 (3)	0.106 (2)	0.0549 (16)	−0.0167 (19)	−0.0024 (17)	−0.0058 (14)
O6	0.148 (3)	0.0690 (15)	0.0753 (17)	0.0389 (16)	0.0009 (16)	−0.0061 (14)
O7	0.095 (2)	0.0828 (18)	0.111 (2)	0.0201 (15)	−0.0245 (17)	0.0184 (15)

### Geometric parameters (Å, º)

**Table:**

C1—O1	1.257 (3)	C10—N6	1.323 (4)
C1—C2	1.438 (4)	C10—C11	1.368 (4)
C1—C6	1.449 (4)	C10—H10	0.9300
C2—C3	1.369 (4)	C11—C12	1.357 (4)
C2—N1	1.461 (4)	C11—H11	0.9300
C3—C4	1.378 (4)	C12—N7	1.321 (4)
C3—H3	0.9300	C12—H12	0.9300
C4—C5	1.380 (4)	N1—O2	1.214 (3)
C4—N2	1.453 (4)	N1—O3	1.216 (3)
C5—C6	1.367 (4)	N2—O4	1.210 (4)
C5—H5	0.9300	N2—O5	1.225 (4)
C6—N3	1.454 (4)	N3—O6	1.216 (3)
C7—N4	1.316 (4)	N3—O7	1.219 (3)
C7—C8	1.370 (4)	N4—N5	1.330 (4)
C7—H7	0.9300	N4—H4A	0.862 (10)
C8—C9	1.352 (5)	N5—H5A	0.865 (10)
C8—H8	0.9300	N6—N7	1.323 (3)
C9—N5	1.335 (4)	N6—H6A	0.861 (10)
C9—H9	0.9300	N7—H7A	0.861 (10)
			
O1—C1—C2	123.9 (3)	C11—C10—H10	124.9
O1—C1—C6	124.2 (3)	C12—C11—C10	105.1 (3)
C2—C1—C6	111.9 (2)	C12—C11—H11	127.5
C3—C2—C1	124.4 (3)	C10—C11—H11	127.5
C3—C2—N1	115.8 (2)	N7—C12—C11	107.7 (3)
C1—C2—N1	119.8 (2)	N7—C12—H12	126.1
C2—C3—C4	119.3 (3)	C11—C12—H12	126.1
C2—C3—H3	120.3	O2—N1—O3	123.3 (3)
C4—C3—H3	120.3	O2—N1—C2	119.2 (2)
C3—C4—C5	120.9 (3)	O3—N1—C2	117.5 (3)
C3—C4—N2	119.0 (3)	O4—N2—O5	123.3 (3)
C5—C4—N2	120.1 (3)	O4—N2—C4	118.9 (3)
C6—C5—C4	119.5 (3)	O5—N2—C4	117.8 (3)
C6—C5—H5	120.3	O6—N3—O7	122.3 (3)
C4—C5—H5	120.3	O6—N3—C6	119.8 (3)
C5—C6—C1	123.9 (3)	O7—N3—C6	117.8 (3)
C5—C6—N3	116.4 (3)	C7—N4—N5	106.9 (3)
C1—C6—N3	119.7 (3)	C7—N4—H4A	131 (5)
N4—C7—C8	110.1 (3)	N5—N4—H4A	122 (5)
N4—C7—H7	125.0	N4—N5—C9	109.7 (3)
C8—C7—H7	125.0	N4—N5—H5A	120 (2)
C9—C8—C7	105.3 (3)	C9—N5—H5A	130 (2)
C9—C8—H8	127.3	C10—N6—N7	106.2 (2)
C7—C8—H8	127.3	C10—N6—H6A	127 (4)
N5—C9—C8	108.0 (3)	N7—N6—H6A	126 (4)
N5—C9—H9	126.0	C12—N7—N6	110.8 (3)
C8—C9—H9	126.0	C12—N7—H7A	128 (2)
N6—C10—C11	110.2 (3)	N6—N7—H7A	121 (2)
N6—C10—H10	124.9		
			
O1—C1—C2—C3	179.5 (3)	C10—C11—C12—N7	0.2 (4)
C6—C1—C2—C3	−0.3 (4)	C3—C2—N1—O2	148.3 (3)
O1—C1—C2—N1	−1.3 (4)	C1—C2—N1—O2	−31.0 (4)
C6—C1—C2—N1	179.0 (2)	C3—C2—N1—O3	−29.2 (4)
C1—C2—C3—C4	2.0 (4)	C1—C2—N1—O3	151.5 (3)
N1—C2—C3—C4	−177.3 (2)	C3—C4—N2—O4	4.1 (5)
C2—C3—C4—C5	−1.3 (4)	C5—C4—N2—O4	−175.5 (3)
C2—C3—C4—N2	179.1 (3)	C3—C4—N2—O5	−176.2 (3)
C3—C4—C5—C6	−1.1 (4)	C5—C4—N2—O5	4.2 (5)
N2—C4—C5—C6	178.5 (3)	C5—C6—N3—O6	152.3 (3)
C4—C5—C6—C1	3.1 (4)	C1—C6—N3—O6	−28.5 (4)
C4—C5—C6—N3	−177.7 (3)	C5—C6—N3—O7	−24.9 (4)
O1—C1—C6—C5	178.0 (3)	C1—C6—N3—O7	154.4 (3)
C2—C1—C6—C5	−2.3 (4)	C8—C7—N4—N5	0.1 (4)
O1—C1—C6—N3	−1.3 (4)	C7—N4—N5—C9	−0.1 (3)
C2—C1—C6—N3	178.5 (2)	C8—C9—N5—N4	0.0 (4)
N4—C7—C8—C9	−0.2 (4)	C11—C10—N6—N7	0.2 (4)
C7—C8—C9—N5	0.1 (4)	C11—C12—N7—N6	−0.1 (4)
N6—C10—C11—C12	−0.2 (4)	C10—N6—N7—C12	−0.1 (4)

### Hydrogen-bond geometry (Å, º)

**Table:**

*D*—H···*A*	*D*—H	H···*A*	*D*···*A*	*D*—H···*A*
N4—H4*A*···N6	0.86 (1)	1.81 (1)	2.663 (3)	173 (7)
N5—H5*A*···O1^i^	0.87 (1)	1.95 (1)	2.789 (3)	163 (3)
N5—H5*A*···O6^i^	0.87 (1)	2.42 (3)	2.961 (4)	121 (3)
N6—H6*A*···N4	0.86 (1)	1.81 (1)	2.663 (3)	174 (7)
N7—H7*A*···O1	0.86 (1)	2.04 (2)	2.864 (3)	160 (3)
N7—H7*A*···O2	0.86 (1)	2.29 (3)	2.841 (3)	122 (3)
C12—H12···O4^ii^	0.93	2.61	3.512 (5)	165

Symmetry codes: (i) *x*+1, *y*, *z*; (ii) −*x*, −*y*+2, −*z*+1.
